# Endogenous ABA alleviates rice ammonium toxicity by reducing ROS and free ammonium via regulation of the SAPK9–bZIP20 pathway

**DOI:** 10.1093/jxb/eraa076

**Published:** 2020-02-17

**Authors:** Li Sun, Dong-Wei Di, Guangjie Li, Herbert J Kronzucker, Xiangyu Wu, Weiming Shi

**Affiliations:** 1 State Key Laboratory of Soil and Sustainable Agriculture, Institute of Soil Science, Chinese Academy of Sciences, Nanjing, Jiangsu, China; 2 State Key Lab of Crop Genetics and Germplasm Enhancement, Cytogenetics Institute, Nanjing Agricultural University/JCIC-MCP, Nanjing, Jiangsu, China; 3 School of Agriculture and Food, University of Melbourne, Parkville, VIC, Australia; 4 Faculty of Land and Food Systems, University of British Columbia, Vancouver, BC, Canada; 5 Key Lab of Plant-Soil Interaction, MOE, College of Resources and Environmental Sciences, China Agricultural University, Beijing, China; 6 Michigan State University, USA

**Keywords:** ABA, ammonium assimilation, antioxidant activity, high ammonium stress, OsbZIP20, OsSAPK9

## Abstract

Ammonium (NH_4_^+^) is one of the principal nitrogen (N) sources in soils, but is typically toxic already at intermediate concentrations. The phytohormone abscisic acid (ABA) plays a pivotal role in responses to environmental stresses. However, the role of ABA under high-NH_4_^+^ stress in rice (*Oryza sativa* L.) is only marginally understood. Here, we report that elevated NH_4_^+^ can significantly accelerate tissue ABA accumulation. Mutants with high (*Osaba8ox*) and low levels of ABA (*Osphs3*-1) exhibit elevated tolerance or sensitivity to high-NH_4_^+^ stress, respectively. Furthermore, ABA can decrease NH_4_^+^-induced oxidative damage and tissue NH_4_^+^ accumulation by enhancing antioxidant and glutamine synthetase (GS)/glutamate synthetasae (GOGAT) enzyme activities. Using RNA sequencing and quantitative real-time PCR approaches, we ascertain that two genes, *OsSAPK9* and *OsbZIP20*, are induced both by high NH_4_^+^ and by ABA. Our data indicate that OsSAPK9 interacts with OsbZIP20, and can phosphorylate OsbZIP20 and activate its function. When OsSAPK9 or OsbZIP20 are knocked out in rice, ABA-mediated antioxidant and GS/GOGAT activity enhancement under high-NH_4_^+^ stress disappear, and the two mutants are more sensitive to high-NH_4_^+^ stress compared with their wild types. Taken together, our results suggest that ABA plays a positive role in regulating the OsSAPK9–OsbZIP20 pathway in rice to increase tolerance to high-NH_4_^+^ stress.

## Teaser

Abscisic acid plays a positive role in regulating the OsSAPK9–OsbZIP20 pathway and elevating antioxidant and GS/GOGAT activities in rice to increase tolerance to high-NH_4_^+^ stress.

## Introduction

Nitrate (NO_3_^−^) and ammonium (NH_4_^+^) are the two major inorganic nitrogen (N) forms accessed by plant roots. Although NH_4_^+^ is frequently absorbed by roots more readily, and its assimilation requires less energy than that of NO_3_^−^, excessive NH_4_^+^ is toxic to plants (Britto *et al.*, 2001; Kronzucker *et al.*, 2001; Britto and Kronzucker, 2002; Li *et al.*, 2014). Under high-NH_4_^+^ stress, the roots, as the site of initial stress perception, undergo a series of physiological, cellular, and morphological changes, including inhibition of root growth and gravitropism (Li *et al.*, 2010; Zou *et al.*, 2012, 2013; Di *et al.*, 2018). Several physiological mechanisms have been proposed to explain NH_4_^+^ toxicity, and these have included rhizosphere acidification, ionic imbalance, carbon metabolism disturbance, energy consumption, and hormone alteration (Britto and Kronzucker, 2002; Di *et al.*, 2018). Many components of the stress syndrome, however, remain unclear.

NH_4_^+^ toxicity takes place when plants accumulate high tissue levels of free NH_4_^+^, resulting from both excessive NH_4_^+^ exposure and disturbance of NH_4_^+^ assimilation in plant cells (Barker and Corey, 1991; Bittsánszky *et al*., 2015). Therefore, the capacity for NH_4_^+^ assimilation, mediated by glutamine synthetase (GS), glutamate synthase (GOGAT), and glutamate dehydrogenase (GDH), is viewed as critical in the detoxification of excessive NH_4_^+^ (Miflin and Habash, 2002; Hoai *et al.*, 2003; Skopelitis *et al.*, 2006). Knockout of the genes encoding these enzymes results in increased free NH_4_^+^ accumulation in plant tissue (Tabuchi *et al.*, 2005; Tamura *et al.*, 2010, 2011; Funayama *et al.*, 2013). Plant species with higher GS activity accumulate less free NH_4_^+^ and are indeed more tolerant to high NH_4_^+^ (Cruz *et al.*, 2006; Omari *et al.*, 2010). A recent study in *Arabidopsis thaliana* revealed that AtNRT1.1 negatively regulates NH_4_^+^ tolerance by inhibiting the activities of GS, GOGAT, and GDH through a nitrate-independent pathway, and knockout of AtNRT1.1 enhances NH_4_^+^ assimilation and reduces free NH_4_^+^ accumulation in *nrt1.1* mutants (Jian *et al.*, 2018). Clearly, therefore, the regulation of NH_4_^+^ assimilation under high NH_4_^+^ warrants further study.

It is well known that reactive oxygen species (ROS) are generated under various environmental stresses, and that high levels of ROS induce oxidative damage and then injury and programmed cell death (Mittler and Blumwald, 2015). Excessive NH_4_^+^ accumulation in plant cells has been reported to induce high levels of H_2_O_2_ and oxidative stress in the roots of *A. thaliana*, tomato, and rice (Patterson *et al.*, 2010; Fernández-Crespo *et al*., 2015; Xie *et al.*, 2015). Moreover, NH_4_^+^ also up-regulates the activities of antioxidant enzymes, including catalase (CAT), glutathione reductase (GR), and superoxide dismutase (SOD), all active in scavenging ROS and relieving oxidative stress (Nimptsch and Pflugmacher, 2007; Wang *et al.*, 2008; Patterson *et al.*, 2010; Xie *et al.*, 2015). Recently, the heme–heme oxygenase OsSE5 has been reported to regulate root growth under high-NH_4_^+^ stress by activating antioxidant enzymes, namely ascorbate peroxidase (APX), CAT, and SOD, and overexpression of OsSE5 in *A. thaliana* increases NH_4_^+^ tolerance (Xie *et al.*, 2015). Concurrent overexpression of *OsGS1.1* and *OsGS2* leads to increased accumulation of glutathione (GSH), a powerful non-enzymatic antioxidant, and reduced ROS accumulation under high NH_4_^+^ (James *et al.*, 2018).

The phytohormone abscisic acid (ABA) plays a pivotal role in coordinating responses to environmental cues (Yamaguchi-Shinozaki and Shinozaki, 2005, 2006). Several studies have suggested a possible interaction between NH_4_^+^ and ABA. In rice, NH_4_^+^ supply enhances ABA content under drought stress, which, in turn, increases water uptake and is associated with increased drought tolerance (Ding *et al.*, 2016). Moreover, the latter report also showed that ABA transport from roots to shoots increased under NH_4_^+^ supply (Peuke *et al.*, 1998). Our previous study in *A. thaliana* identified a plastid metalloprotease AMOS1/EGY1 as an important intersection point of NH_4_^+^ and ABA (Li *et al.*, 2012). Transcriptome analysis shows that 90% of NH_4_^+^-activated genes are regulated by AMOS1/EGY1, and a large portion of them carry a core motif of an ABA-responsive element in their promoters (Li *et al.*, 2012). Thus, the ABA signaling pathway is deeply involved in the NH_4_^+^ response.

Under environmental stress, ABA content is elevated, which consequently activates the ABA signaling pathway and triggers physiological reactions affecting stress resistance (Mittler and Blumwald, 2015). ABA binds to the receptor RCAR/PYR/PYLS, and then binds to protein phosphatase 2C (PP2C), inhibiting its enzymatic activity and dissociating the PP2C–SNF1-related protein kinase 2 (SnRK2) complex. Subsequently, auto-phosphorylated SnRK2s can activate downstream transcription factors (TFs) and then induce transcription of key genes including those coding for ion channels and late embryogenesis abundant (LEA) proteins (Ma *et al.*, 2009; Soon *et al.*, 2012; Zhang *et al.*, 2019). Of the regulated TFs, the basic leucine zipper (bZIP) TF family has been investigated with special attention in rice and Arabidopsis, due to the synergistic regulation between ABA and environmental stress (Kim, 2006; Nijhawan *et al.*, 2008). In rice, there are 89 bZIPs, and these have been classified into 13 groups (A, B, C, D, E, F, G, H, I, J, K, L, and S) (Nijhawan *et al.*, 2008). Of these, OsZIP66 (Hobo *et al.*, 1999), OsbZIP23 (Xiang *et al.*, 2008), OsbZIP46 (Tang *et al.*, 2012), OsbZIP72 (Lu *et al.*, 2009), OsbZIP12/OsABF1 (Hossain *et al.*, 2010), OsABI5 (Zou *et al.*, 2008), and OsbZIP71 (Liu *et al.*, 2012) have been reported to regulate osmotic stress responses via ABA signal transduction. Nevertheless, whether the interaction of ABA signaling and OsbZIP is involved in the response to high-NH_4_^+^ stress remains unclear.

Here, we analyzed gene expression, enzyme activities, metabolites, and the physiological processes in ABA-related mutants and their wild types under high-NH_4_^+^ stress. We aimed to explore: (i) whether ABA functions in response to high-NH_4_^+^stress; (ii) which physiological processes are involved; and (iii) which members of the ABA signaling pathway are involved. Our results provide new understanding of the involvement of ABA signaling in response to high-NH_4_^+^ stress.

## Materials and methods

### Plant materials and growth conditions

Seeds were surface-sterilized with 1% sodium hypochlorite for 10 min, washed extensively with distilled water, and then germinated in distilled water at 28 °C for 2 d. The treatment solution was applied as described in Sun *et al.* (2017), and the solutions containing 1 mM and 7.5 mM (NH_4_)_2_SO_4_ were designated as normal ammonium (NA) and high ammonium (HA), respectively. Solutions were exchanged every 12 h to ensure that plants remained at a nutritional steady state in the hydroponic system. Samples (three biological replicates) of roots were taken after the imposition of N excess treatments 12 h later, frozen immediately, and stored at –80 °C for associated analyses.

### Determination of H_2_O_2_ content

Determination of H_2_O_2_ content was performed according to Liu *et al.* (2010). Roots used in measurements of H_2_O_2_ content were stored in liquid nitrogen immediately after harvesting. H_2_O_2_ concentrations were calculated using a standard curve prepared with known concentrations of H_2_O_2_.

### Free NH_4_^+^ content determination

Roots were collected and desorbed with 10 mM CaSO_4_ for 5 min, to remove extracellular NH_4_^+^. Free NH_4_^+^ contents were determined as previously described (Sun *et al.*, 2017).

### Determination of glutamine and glutamate contents

A 0.5 g aliquot (FW) of roots was frozen in liquid nitrogen immediately after each treatment. Then, 10 ml of 50% ethanol solution (containing 0.01 mM HCl) was added and placed in a water bath at 4 °C and subjected to ultrasound for 30 min. Then, centrifugation took place at 12 000 rpm at 4 °C for 5 min, and ~1 ml of extract was filtered using a 0.22 µm filter membrane and then placed in a SYKAM Amino Acid Analyzer for further analysis (Sykam, Germany).

### Malondialdehyde (MDA) contents and antioxidant enzyme assays

MDA contents were measured according to the method of Heath and Packer (1968). The SOD activity in roots was estimated by monitoring the inhibition of the photochemical reduction of nitroblue tetrazolium (NBT) according to the method of Giannopolitis and Ries (1977). CAT activity of root was assayed from the rate of H_2_O_2_ decomposition as measured by the decrease of absorbance at 240 nm, following the procedure of Aebi (1984). APX activity in roots was assayed according to Chance and Maehly (1955), and the activity was determined by monitoring the increase of absorbance at 470 nm.

### Estimation of proline and soluble sugar contents

Root samples (1 g) were extracted in 3% sulfosalicylic acid, and the proline content was estimated following the method of Dey *et al.* (2016), using acid-ninhydrin reagent. The proline content was calculated from a standard curve prepared against l-proline (0–100 μg). Total soluble sugar content was estimated following the method described by Dey *et al.* (2016). The total sugar content was estimated from a standard curve plotted using 0–100 μg of glucose.

### NH_4_^+^ assimilation enzyme analysis

For the GS, GOGAT, and GDH enzyme activities, crude root extracts were collected and enzyme activities were determined according to previous methods (Jian *et al.*, 2018).

### Transactivation assay

The transactivation assay was performed according to the methods described by Hossain *et al.* (2010). The yeast strain Y2HGold (Clontech) was used to test for the presence of an activation domain in the gene. For the transcription activation assay, sequences containing the full-length cDNA of *OsbZIP20* were fused in-frame with *p*GBKT7 to construct *p*GBKT7-OsbZIP20. The construct was transformed into the yeast strain Y2HGold. The empty pGBKT7 vector was also transformed into the yeast cells as a negative control. The transformants were incubated on SD/Leu-/Trp-/His+2 mM 3-aminotriazole (3-AT) at 30 °C for 2–3 d.

### Yeast one-hybrid assay

To analyze the G-box- or ABRE-binding activity of OsbZIP20, the construct was inserted into *p*HIS2 vectors (Clontech). In addition to the HIS2 minimal promoter in the *p*HIS2 expression vector, we synthesized oligonucleotide sequences that fused four tandem repeat copies of the G-box (5'-CACGTG-3') or ABRE (5'-GTACGTGTC-3'). This G-box or ABRE was annealed and ligated to form four tandem copies and inserted into the *p*HIS2 vector that had been digested with the same enzymes, and the fusion construct was *p*HIS2::G-box or *p*HIS2::ABRE. The full-length cDNA of OsbZIP20 was synthesized and introduced into *p*GADT7 (Clontech) to construct the *p*GADT7-OsbZIP20 vector. These plasmids (*p*GADT7-OsbZIP20 and *p*HIS2::G-box or *p*HIS2::ABRE) were transformed into the yeast strain Y2HGold (Clontech) carrying the reporter gene HIS2 and used for the binding experiment with yeast. Samples were grown on selective medium plates SD/Leu-/Trp-/His+2 mM 3-AT for 2–3 d at 30 °C.

### Two-hybrid system assay

The full-length cDNAs of OsbZIP20 and OsSAPK8/9/10 were synthesized and introduced into *p*GADT7 and *p*GBKT7, respectively (Clontech). The plasmids *p*GBKT7 and *p*GADT7 were each transformed into Y2HGold acting as the control. *p*GBKT7-OsSAPK8/9/10+*p*GADT7-OsbZIP20 were transformed into Y2HGold. Empty pGADT7 and *p*GBKT7 vectors were also transformed into yeast cells as negative controls. The transformants were incubated on SD/Leu-/Trp-/His+2 mM 3-AT at 30 °C for 2–3 d.

### BiFC analyses

The full-length cDNAs of OsbZIP20 and OsSAPK9 without stop codons were synthesized and introduced into serial pGreen-pSAT1 vectors containing either N- or C-terminal enhanced yellow fluorescent protein (eYFP) fragments and introduced into *Agrobacterium* as described previously (Hou *et al.*, 2010). Three-week-old *Nicotiana benthamiana* leaves were agroinfiltrated with agrobacterial cells containing the indicated constructs. Two days after incubation, fluorescence was analyzed by confocal microscopy.

### In vitro kinase assay

The *in vitro* kinase assay was performed according to the methods described by Dey *et al.* (2016). The recombinant pRSET plasmid (Invitrogen) containing the 1086 bp coding sequence (CDS) of OsSAPK9 in *Xho*I and *Eco*RI restriction sites allowed expression of 6×His N-terminal tagged OsSAPK9 protein in *Escherichia coli* BL21 (DE3) pLysE strain (Invitrogen) upon induction by 1 mM isopropyl-β-d-thiogalactopyransode (IPTG). Similarly, first the 893 bp (encoding 297 amino acids) from the OsbZIP20 CDS of *Oryza rufipogon* were cloned into a pRSET vector in *Bam*HI and *Eco*RI restriction sites, transformed, and expressed in pLysE cells with induction by 0.5 mM IPTG. The expressed proteins were purified in the native condition and used for the *in vitro* kinase assay. *In vitro* phosphorylation of the generic substrate histone III (Sigma) was performed as described previously. *In vitro* phosphorylation of OsbZIP20 was performed by incubating the individual reaction mixture for 40 min at 25 °C following the above-mentioned protocol. The products were fractionated by 12% SDS–PAGE and visualized by autoradiography.

### Quantitative real-time PCR

Total RNA was extracted from shoots and roots harvested at the specified time points with TRIzol reagent (Invitrogen, USA) and treated with RNase-free DNase I (Promega). Total RNA (2 μg) was used for reverse transcription with M-MLV Reverse Transcriptase (Promega), and the cDNA samples were diluted 2-fold. For quantitative real-time PCR (qRT-PCR), triplicate quantitative assays were performed on each cDNA dilution with ChamQ SYBR qPCR MasterMix (Q311-02,Vazyme Biotech Co., Ltd), and a CFX Manager sequence detection system according to the following protocol: denaturation at 95 °C for 30 s for initiation; denaturation at 95 °C for 10 s; and 40 cycles of amplification, annealing, and extension at 55 °C/60 °C for 30 s. The specificity of the amplification was ascertained using a melting curve performed from 65°C to 95 °C, as well as sequencing of the amplification. Three independent replicates were performed per experiment, and the means and corresponding SEs were calculated. The *OsActin1* gene was used as a normalization control. Primer sequences are as listed in Supplementary Table S1 at *JXB* online).

### Phos-tag SDS–PAGE assays

The peptide fragment (CTGLDYAGDDPFTGLSP) of bZIP20 was used to immunize rabbits, and antiserum was collected to determine the titer. For phos-tag SDS–PAGE assays, the nuclear protein of roots (200 mg FW) was obtained using a Nuclear Extraction Kit (BB-3154-1, BestBio). Nuclear protein was then used for phos-tag SDS–PAGE by the SuperSep™ Phos-tag™ Kit (198-17981, Wako). All experiments were completed following the supplier’s operation manual. Coomassie Brilliant Blue staining was use to normalize protein amounts.

### Statistical analysis

All statistical analyses were performed using Prism 6 software (GraphPad Software), and one-way or two-way ANOVA was performed. *P*<0.05 was set as the significance cut-off. All values were presented as means ±SD.

## Results

### Higher endogenous ABA enhances NH_4_^+^ tolerance in rice

**Fig. 1.**
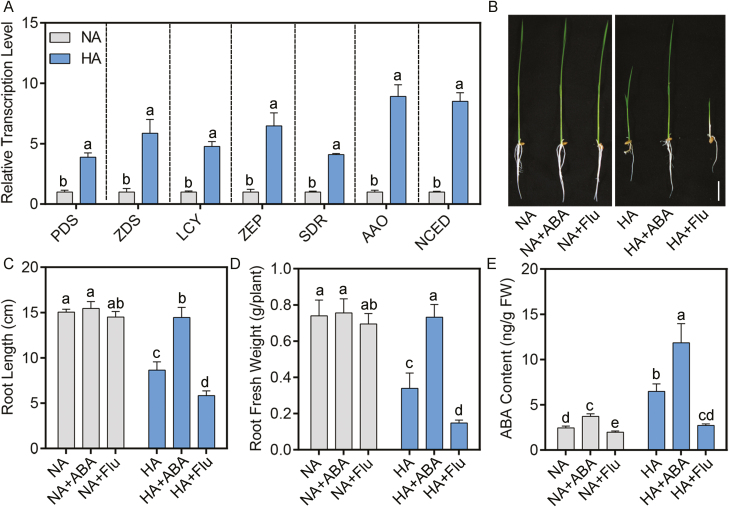
Exogenous ABA relieves toxicity of high NH_4_^+^ in rice. (A) The regulation of ABA biosynthesis genes in rice under 7.5 mM (NH_4_)_2_SO_4_ (high-NH_4_^+^, HA) treatment; 5-day-old seedling were transferred to HA medium for another 12 h, and then roots were collected for RNA extraction and qPCR analysis. Data are the means of three biological replicates. (B–E) root phenotypes (B); maximum primary root length (C); root fresh weight (D); and ABA contents of roots (E) grown in normal-NH_4_^+^ (NA) and HA medium with 1 μM ABA or 0.2 μM fluorine (Flu). Five-day-old seedlings were transferred to treatment medium for another 10 d (*n*=10). Scale bar=5 cm. Data are analyzed by two-way ANOVA following Duncan’s test. Error bars with different letters represent a statistical difference (*P*<0.05, Duncan’s test).

Our previous RNA sequencing (RNA-seq) analysis showed that the transcript levels of ABA biosynthesis genes in roots were up-regulated by high NH_4_^+^ (Sun *et al.*, 2017). Here, we show, using qRT-PCR, that genes involved in ABA biosynthesis were up-regulated 3.5- to 9.7-fold following high-NH_4_^+^ treatment (Fig. 1A). To investigate the role of ABA under high-NH_4_^+^ stress in rice, a pharmacological investigation was carried out by using exogenous ABA and fluorine (Flu; ABA biosynthesis inhibitor), and then root length and fresh weight were determined (Fig. 1B–D). Our results reveal that maximum primary root length and root fresh weight when grown in high-NH_4_^+^ media were 57.5% and 45.8% of those grown on control (1 mM NH_4_^+^) media; they were 93.5% and 96.8% following addition of ABA, and 40.1% and 21.3% after addition of Flu (Fig. 1B–D). Moreover, measurements of ABA content also showed that high NH_4_^+^ promoted ABA accumulation, and that ABA and Flu could increase or decrease this promotion (Fig. 1E). These results indicate that high NH_4_^+^ stimulates ABA biosynthesis and that ABA plays a positive role under high-NH_4_^+^ stress.

**Fig. 2.**
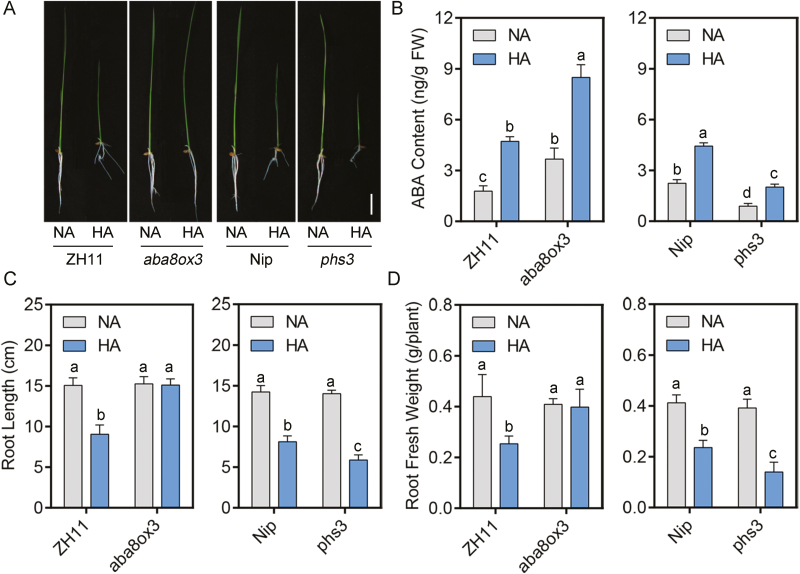
Higher endogenous ABA enhances NH_4_^+^ tolerance in rice. (A–D) Root phenotypes (A); ABA contents in roots (B); maximum primary root length (C); and root fresh weight (D) of *aba8ox3*, *phs3*, and their backgrounds ZH11 and Nip, grown in normal-NH_4_^+^ (NA) and high-NH_4_^+^ (HA) medium. Five-day-old seedlings were transferred to treatment medium for another 10 d (*n*=10). Scale bar=5 cm. Data are analyzed by two-way ANOVA following Duncan’s test. Error bars with different letters represent a statistical difference (*P*<0.05, Duncan’s test).

To further examine the function of ABA, a mutant high in endogenous ABA, *aba8ox3*, and a mutant low in endogenous ABA, *phs3*, were used (Fig. 2A; Supplementary Fig. S1) (Fang *et al.*, 2008; Cai *et al.*, 2015). Endogenous ABA content determination showed that *aba8ox3* and *phs3* contained more or less ABA compared, respectively, with their backgrounds under control and high-NH_4_^+^ conditions (Fig. 2B). Similar to ABA or Flu supplementation, the relative maximum primary root length and root fresh weight under the high-NH_4_^+^ treatment in *aba8ox* (98.9% and 97.3%) and in *phs3* (41.9% and 35.7%) were higher or lower, respectively, compared with their backgrounds (60% and 57.8% in ZH11; 57.1% and 57.2% in Nip) (Fig. 2). To sum up, ABA plays a protective role in the root growth inhibition brought about by high NH_4_^+^.

### Endogenous ABA improves NH_4_^+^ tolerance by strengthening NH_4_^+^ assimilation

**Fig. 3.**
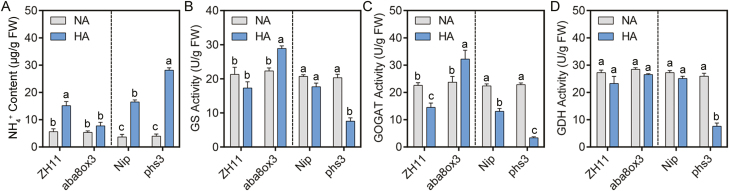
ABA improves the activities of NH_4_^+^ assimilation enzymes. (A–D) NH_4_^+^ contents (A); GS activity (B); GOGAT activity (C); and GDH activity (D) of *aba8ox3*, *phs3*, and their backgrounds ZH11 and Nip, grown in normal-NH_4_^+^ (NA) and high-NH_4_^+^ (HA) medium. Five-day-old seedlings were transferred to treatment medium for another 10 d. Data are analyzed by two-way ANOVA following Duncan’s test (*n*=3). Error bars with different letters represent a statistical difference (*P*<0.05, Duncan’s test).

To dissect the reason for improved NH_4_^+^ tolerance by increasing endogenous ABA, we first determined free NH_4_^+^ contents in roots. Our data show that high NH_4_^+^ induces free NH_4_^+^ accumulation in wild-type roots, and that it increases in *phs3* and decreases in *aba8ox3* (Fig. 3A). Subsequently, we tested the enzyme activities of GS, GOGAT, and GDH under control and high NH_4_^+^. The activities of these enzyme exhibited no differences between *aba8ox3*, *phs3*, and their backgrounds under control conditions (Fig. 3B–D). However, when examining relative GS, GOGAT, and GDH activities (high NH_4_^+^/control), activities were 129.4, 135.6, and 93.3% in *aba8ox3*, and 37.2%, 14.7, and 29.3% in *phs3* compared with the background ZH11 (81.1, 64.2, and 85.4%) and Nip (85.5, 58.3, and 92.0%), respectively (Fig. 3B–D). A similar promotion or inhibition of enzyme activities involved in NH_4_^+^ assimilation was observed when exogenous ABA or Flu were applied (Supplementary Fig. S2). Overall, the data indicate that ABA can accelerate NH_4_^+^ assimilation by activating the enzyme activities of GS, GOGAT, and GDH under high-NH_4_^+^ stress and that root growth can be maintained under high-NH_4_^+^ stress.

### Higher endogenous ABA reduces cell damage by up-regulating antioxidant activities at high NH_4_^+^

**Fig. 4.**
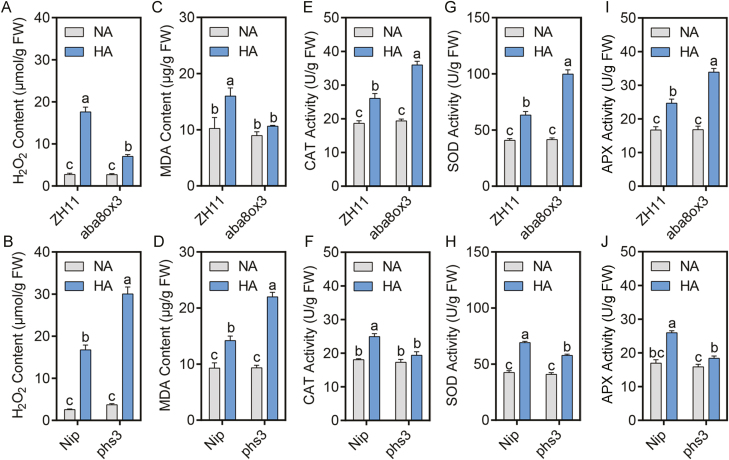
Higher endogenous ABA up-regulates antioxidant activities under high-NH_4_^+^ stress. (A and B) H_2_O_2_ contents; (C and D) MDA contents; (E and F) CAT activity; (G and H) SOD activity; and (I and J) APX activity of *aba8ox3* and its background ZH11, and *phs3* and its background Nip, grown in normal-NH_4_^+^ (NA) and high-NH_4_^+^ (HA) medium. Five-day-old seedlings were transferred to treatment medium for another 10 d and then roots were collected for examination. Data are analyzed by two-way ANOVA following Duncan’s test (*n*=3). Error bars with different letters represent a statistical difference (*P*<0.05, Duncan’s test).

Stresses are frequently associated with the generation of ROS, such as H_2_O_2_, and ROS build-up, in turn, leads to lipid peroxidation, which results in the production of MDA (Dey *et al.*, 2016). MDA is a stress-specific molecular marker that is indicative of the extent of membrane injury and cell and tissue damage. To determine whether high-NH_4_^+^ stress also induces ROS generation and whether ABA is involved in this, we measured H_2_O_2_ and MDA contents in *aba8ox3*, *phs3*, and their backgrounds under control and high-NH_4_^+^ stress. Our data show increases in H_2_O_2_ and MDA contents in all tested materials under high NH_4_^+^. However, increases were less in *aba8ox3* and more in *phs3* after NH_4_^+^ treatment, indicating that higher endogenous ABA can reduce oxidative damage by decreasing ROS content under NH_4_^+^ stress (Fig. 4A–D).

Furthermore, we also determined the enzyme activities of several antioxidant enzymes, including SOD, APX, and CAT, in *aba8ox3*, *phs3*, and their backgrounds under control and high-NH_4_^+^ stress. Our results show that the SOD, APX, and CAT enzyme activities can be induced in the wild types (ZH11 and Nip) by high NH_4_^+^. This induction was strengthened in *aba8ox3*, and was weakened in *phs3* (Fig. 4E–J). Taken together, the data clearly reveal that ABA can reduce oxidative damage induced by ROS by increasing antioxidant enzyme activities.

### Endogenous ABA up-regulates *OsbZIP20* under high-NH_4_^+^ stress

**Fig. 5.**
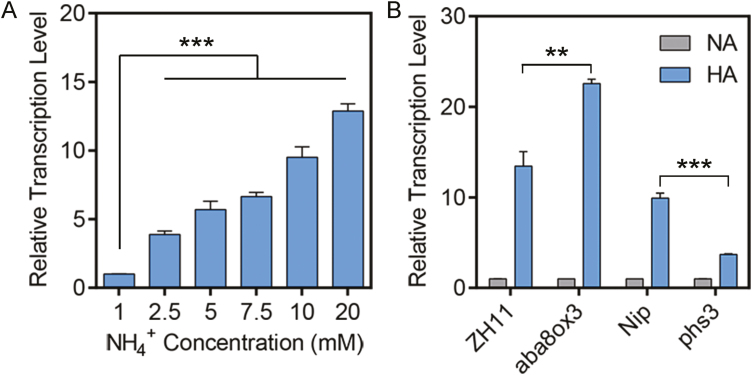
*OsbZIP20* is involved in the response to high NH_4_^+^. (A) Transcript levels of OsbZIP20 in Nip under different concentrations of NH_4_^+^; 5-day-old seedling were transferred to media containing different concentrations of NH_4_^+^ (1, 2.5, 5, 7.5, 10, and 20 mM) for another 12 h, and then roots were collected for RNA extraction and qPCR analysis. (B) Transcript levels of OsbZIP20 in *aba8ox3*, *phs3*, and their backgrounds ZH11 and Nip under high-NH_4_^+^ (HA) conditions. Five-day-old seedling were transferred to normal-NH_4_^+^ (NA) and HA media for another 12 h, and then roots were collected for RNA extraction and qPCR analysis. Data are the means of three biological replicates; Error bars indicate ±SD. **P<0.01 and ***P<0.001 (*t*-test).

Our previous reports revealed that a key gene involved in ABA signaling, namely *OsbZIP20*, was up-regulated by high NH_4_^+^ (Sun *et al.*, 2017). To further unravel the function of endogenous ABA under high NH_4_^+^, we analyzed the transcription of *OsbZIP20* under NH_4_^+^ stress by qRT-PCR. Our data show that the relative transcription levels of *OsbZIP20* under high NH_4_^+^ were 6-fold higher compared with control, and transcription was further induced at higher concentrations of NH_4_^+^ (Fig. 5A). Moreover, the induction of *OsbZIP20* was increased in the *aba8ox3* mutant, and was decreased in the *phs3* mutant (Fig. 5B). These results indicate that *OsbZIP20* is involved in the response to high-NH_4_^+^ stress by an ABA-dependent pathway.

### OsbZIP20 combines with the G-box and ABREs and interacts with OsSAPK9 *in vitro* and *in vivo*

**Fig. 6.**
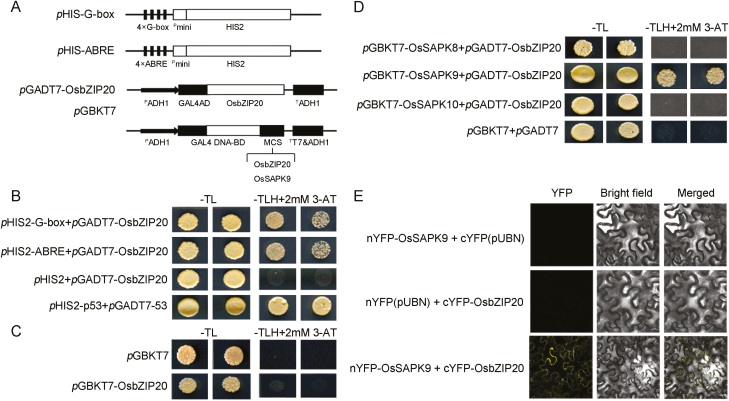
OsSAPK9 and OsbZIP20 function together under high-NH_4_^+^ stress. (A) Illustration of the constructs used in this analysis. 4×G-box and 4×ABRE were each cloned into the pHIS2 vector. Full-length OsbZIP20 was cloned into the vector. The full-length coding sequences of OsbZIP20 and OsSAPK9 were cloned into pGADT7 and pGBKT7 vectors, respectively. (B) G-box- and ABRE-binding activity analysis of OsbZIP20, using the yeast one-hybrid system. Co-transformants of pGADT7-OsbZIP20+pHIS2:G-box and pGADT7- OsbZIP20+pHIS2:ABRE growing on SD/Leu-/Trp-/His+2 mM 3-AT plates. The pGADT7 empty vector was used as the negative control. (C) Transactivation activity analysis of OsbZIP20. Transformants in the yeast strain Y2HGold grown on SD/Leu-/Trp- and SD/Leu-/Trp-/His+2 mM 3-AT. pGBKT7 was used as the negative control (D). Analysis of OsbZIP20 and OsSAPK8/9/10 interaction *in vitro*. Yeast strain Y2HGold transformed with different constructs growing on SD/Leu-/Trp-/His+2 mM 3-AT plates. pGBKT7-OsSAPK8/9/10+pGADT7-OsbZIP20 were transformed into Y2HGold on SD/Leu-/Trp-/His+2 mM 3-AT plates. pGBKT7+pGADT7 were used as the negative control; (E) BiFC analysis of OsSAPK9 and OsbZIP20 interaction in tobacco cells.

To test whether OsbZIP20 is an effective OsbZIP TF, the yeast one-hybrid (Y1H) system was used to determine the transactivation activity of OsbZIP20. The AREB/ABF TF-binding sites, ABRE (ACGTGG/TC) and G-box (CACGTG), were introduced into the *p*HIS2 vector, and the full-length coding sequence of OsbZIP20 was introduced into the *p*GADT7 vector (Fig. 6A). The yeast strain Y1HGold co-transformed with *p*HIS2-G-box or *p*HIS2-ABRE+*p*GADT7-OsbZIP20 still grew well, whereas the yeast cells co-transformed with *p*HIS2-G-box or *p*HIS2-ABRE+*p*GADT7 (negative controls) failed to grow on SD/Leu-/Trp-/His+2 mM 3-AT plates (Fig. 6B). The results indicate that OsbZIP20 can bind to the G-box and ABREs in yeast. Then, we used the yeast two-hybrid (Y2H) system to identify the transactivation activity of OsbZIP20. The transformants grew well on the SD/Trp-/Leu- plates (Fig. 6C). However, when transferred to SD/Leu-/Trp-/His+2 mM 3-AT plates, both *p*GBKT7-OsbZIP20 and negative control failed to grow, indicating that OsbZIP20 had no transactivation activity (Fig. 6C).

The activation of many AREB/ABF proteins requires the phosphorylation by SnRK2 (also referred to as ABA-activated protein kinase, SAPK, in rice) protein kinases (Fujita *et al.*, 2013). To identify the interaction between OsbZIP20 and OsSAPKs in plants, we then performed Y2H system assays. Our results show that, when cultured on the SD/Leu-/Trp-/His+2 mM 3-AT plates, only transformants of *p*GBKT7-OsSAPK9+*p*GADT7-OsbZIP20 grew well compared with *p*GBKT7-OsSAPK8+*p*GADT7-OsbZIP20, *p*GBKT7-OsSAPK10+*p*GADT7-OsbZIP20, and the negative control (Fig. 6D). To confirm the interaction, bimolecular fluorescence complementation (BiFC) analyses were performed in tobacco cells (Fig. 6E). Our data show that YFP was reconstituted when the CDSs of OsSAPK9 and OsbZIP20 were co-expressed (Fig. 6E). In contrast, OsSAPK9–nYFP and cYFP, or nYFP and cYFP–OsbZIP20, did not result in fluorescence (Fig. 6E), suggesting that OsSAPK9 and OsbZIP20 interaction is specific. These results suggested that only OsSAPK9 could interact with OsbZIP20 *in vivo* and *in vitro*.

### OsSAPK9 can phosphorylate OsbZIP20 *in vivo* and *in vitro*

**Fig. 7.**
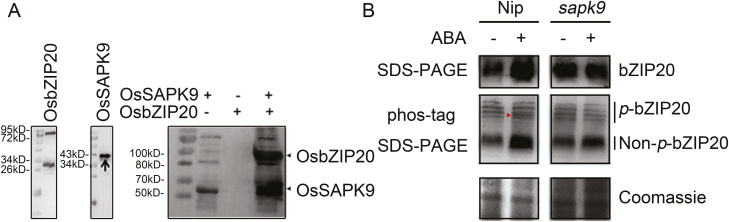
OsSAPK9 is involved in phosphorylation of OsbZIP20 *in vivo* and *in vitro.* (A) Autoradiographs showing *in vitro* phosphorylation of the OsbZIP20 substrate with recombinant OsSAPK9. Coomassie Blue-stained SDS–PAGE showing *E. coli*-expressed recombinant His-tagged SAPK9 and His-tagged OsbZIP20 protein purified through Ni-NTA chromatography under native conditions. (B) ABA changes the phosphorylation of OsbZIP20 in an SAPK9-dependent manner. Fourteen-day-old seedlings were transferred to new solution with or without 1 μM ABA for another 12 h, and then roots were collected for nuclear protein extraction. A red arrow indicates the new phosphorylation pattern of OsbZIP20 induced by exogenous ABA.

**Fig. 8.**
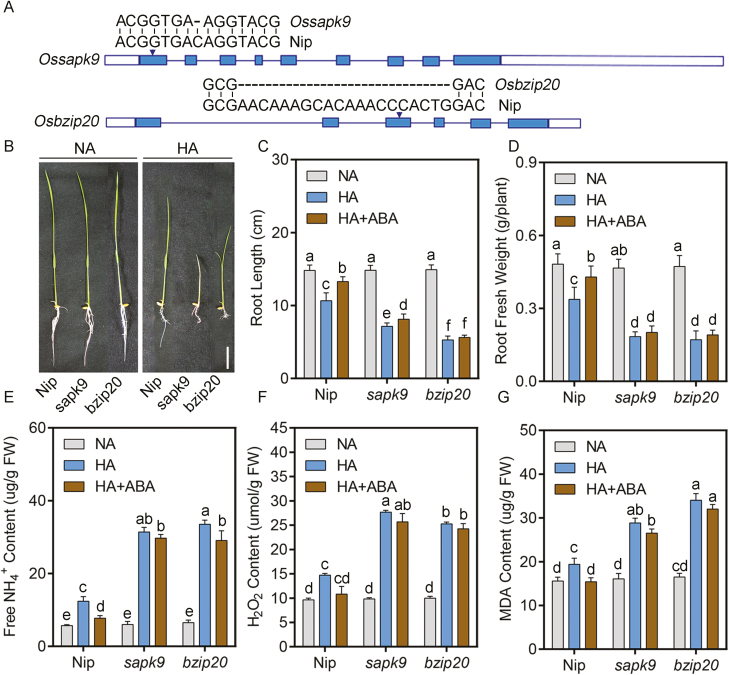
OsSAPK9 and OsbZIP20 knockout increases sensitivity to high NH_4_^+^. (A) Mutation sites of *sapk9* and *bzip20* mutants generated by CRISPR/Cas9; (B) Phenotypes of *sapk9* and *bzip20* grown on normal-NH_4_^+^ (NA) and high-NH_4_^+^ (HA) medium; (C and D) maximum primary root length (C); and root fresh weight (D). Five-day-old seedlings were transferred to treatment medium for another 10 d (*n*=10). (E) Free NH_4_^+^ contents; (F) H_2_O_2_ contents; and (G) MDA contents of *sapk9*, *bzip20*, and Nip grown in NA and HA media. Five-day-old seedling were transferred to NA and HA media for another 10 d, and then roots were collected for examination. Scale bar=5 cm. Data are analyzed by two-way ANOVA following Duncan’s test (*n*=3). Error bars with different letters represent a statistical difference (*P*<0.05, Duncan’s test).

To further investigate how OsSAPK9 interacts with OsbZIP20, we purified the OsSAPK9 and OsbZIP20 proteins in the *E. coli* system (Fig. 7A). Our results verified that OsSAPK9 can phosphorylate OsbZIP20 (Fig. 7A). Furthermore, to investigate the role of ABA in the phosphorylation of OsbZIP20 by OsSAPK9, we analyzed the phosphorylation of bZIP20 by phos-tag SDS–PAGE in Nip and in the OsSAPK9 knockout mutant (Figs 7B, 8A). Our results show that ABA can induce the expression of OsbZIP20, and a new phosphorylated band of OsbZIP20, and that these inductions do not occur in the knockout mutant *sapk9* (Figs 7B, 8A). Overall, these data reveal that the activity of OsbZIP20 can be activated by the phosphorylation of OsSAPK9 and that ABA can promote this phosphorylation in an OsSAPK9-dependent manner.

### bzip20 and sapk9 mutants show higher sensitivity to high NH_4_^+^, accompanied by higher free NH_4_^+^ and H_2_O_2_ accumulation in tissue

To further analyze OsbZIP20 and OsSAPK9 function under high-NH_4_^+^ stress, knockout mutants (*sapk9* and *bzip20*) were constructed by clustered regularly interspaced palindromic repeats (CRISPR)/CRISPR-associated protein 9 (Cas9) (Fig. 8A). Sequence analysis showed there was a single base C deletion in the first exon of *sapk9* and a 20 base deletion in the third exon of *bzip20*, respectively, which resulted in gene inactivation (Fig. 8A). We then observed the phenotypes of *sapk9* and *bzip20* under control and high-NH_4_^+^ conditions. There was no difference among Nip, *sapk9*, and *bzip20* under control conditions, whereas the high NH_4_^+^ inhibited root growth in all genotypes, with greater inhibition in *sapk9* and *bzip20* (Fig. 8B–D). Exogenous ABA could rescue root growth in Nip, but not in *sapk9* and *bzip20* (Fig. 8C, D). These results indicate that OsSAPK9 and OsbZIP20 were involved in high NH_4_^+^ response via an ABA-dependent pathway.

To further test the roles of OsSAPK9 and OsbZIP20 in response to high-NH_4_^+^ stress, we then measured the free NH_4_^+^ content in all genotypes under control and high NH_4_^+^ conditions. All genotypes grown in control solution exhibited similar free NH_4_^+^ accumulation. However, the free NH_4_^+^ accumulation increased 1.18-fold in Nip, 4.23-fold in *sapk9*, and 4.15-fold in *bzip20* compared with the control condition (Fig. 8E). With the addition of exogenous ABA to high-NH_4_^+^ solutions, compared with the high-NH_4_^+^ condition, the free NH_4_^+^ accumulation decreased 37.7%, but only 5.4% and 13.4% in *sapk9* and *bzip20*, respectively (Fig. 8E).

H_2_O_2_ contents in roots varied slightly among the Nip and mutants in the control condition and were 0.53, 1.81, and 1.52 times higher in Nip, *sapk9*, and *bzip20*, respectively, under high NH_4_^+^ compared with control (Fig. 8F). Consistently, the MDA concentrations were also increased in *sapk9* and *bzip20* compared with Nip under high-NH_4_^+^ stress (Fig. 8G). Exogenous ABA inhibited the accumulation of H_2_O_2_ and MDA in Nip roots, but not in *sapk9* and *bzip20* roots (Fig. 8F, G). To sum up, OsSAPK9 and OsbZIP20 were involved in the high-NH_4_^+^ response through regulating free NH_4_^+^ and H_2_O_2_ accumulation in roots.

### OsSAPK9 and OsbZIP20 function under high-NH_4_^+^ stress by enhancing NH_4_^+^ assimilation and antioxidant activity

**Fig. 9.**
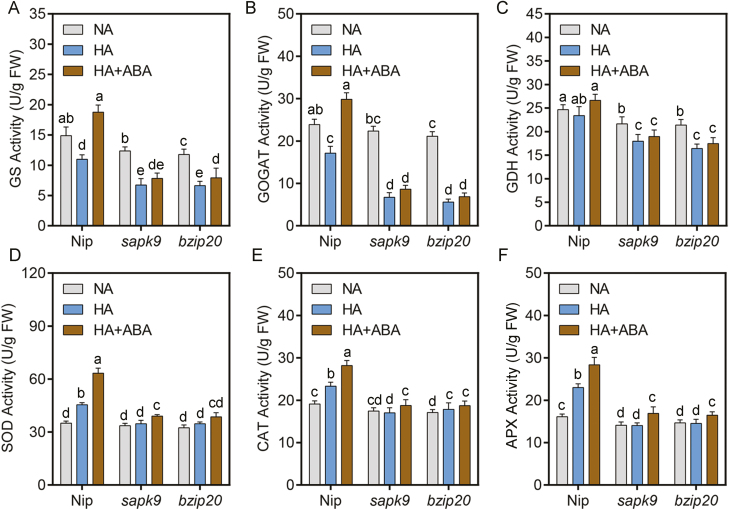
The OsSAPK9–OsbZIP pathway is involved in high-NH_4_^+^ stress by up-regulating NH_4_^+^ assimilation and antioxidant activity. The activities of GS (A), GOGAT (B), GDH (C), SOD (D), CAT (E), and APX (F). Five-day-old seedlings were transferred to treatment medium for another 10 d, and then roots were collected for examination. Data are analyzed by two-way ANOVA following Duncan’s test (*n*=3). Error bars with different letters represent a statistical difference (*P*<0.05, Duncan’s test).

Under control conditions, GS and GOGAT activities were slightly lower in *sapk9* and *bzip20* than in Nip, in roots (Fig. 9A, B). The GS and GOGAT activities decreased in Nip roots under high-NH_4_^+^ stress and more so in *sapk9* and *bzip20* mutants under the same high-NH_4_^+^ conditions (Fig. 9A, B). The activity of GDH was slightly higher in Nip than in *sapk9* and *bzip20* roots under control conditions (Fig. 9C). High NH_4_^+^ reduced GDH activity by 5.2% in Nip but to a greater extent in *sapk9* and *bzip20*, where it decreased by 17.1% and 23.3%, respectively (Fig. 9C). Consistently, the accumulation of Glu and Gln induced by high NH_4_^+^ was also reduced in the *sapk9* and *bzip20* mutants (Supplementary Fig. S3).

Only mild differences in SOD, CAT, and APX activities were observed in roots of Nip, *sapk9*, and *bzip20* (Fig. 9D–F). However, the activities of SOD, CAT, and APX were increased 29.7, 21.9, and 42.6% in Nip under high-NH_4_^+^ conditions, respectively, while they were not increased in *sapk9* and *bzip20* (Fig. 9D–F). Furthermore, exogenous ABA application can obviously promote the activities of these enzymes, including GS, GOGAT, GDH, SOD, CAT, and APX under high-NH_4_^+^ stress in Nip, and this promotion was reduced or even disappeared in *sapk9* and *bzip20* (Fig. 9). Taken together, these results suggest that OsSAPK9 and OsbZIP20 have a positive role in the activities of NH_4_^+^ assimilation and antioxidant enzymes in response to high-NH_4_^+^ stress via an ABA-dependent pathway.

## Discussion

### ABA is involved in the response to NH_4_^+^ stress by enhancing NH_4_^+^ assimilation and antioxidant enzyme activities

The plant hormone ABA plays a key role in a broad array of adaptive stress responses to environmental stimuli in plants (Cutler *et al.*, 2010; Raghavendra *et al.*, 2010; Weiner *et al.*, 2010). Many studies have revealed that NH_4_^+^ can regulate ABA biosynthesis and transport (Peuke *et al.*, 1998; Li *et al.*, 2012; Ding *et al.*, 2016). In *A. thaliana*, transcriptome analysis revealed that many genes induced by high NH_4_^+^ contain the core motif of the ABARE in their promoters, indicating that ABA signaling is involved in the response to high NH_4_^+^ (Li *et al.*, 2012). However, how ABA signaling regulates NH_4_^+^ stress in rice has remained unknown. Here, we identify that ABA positively regulates NH_4_^+^ tolerance. This conclusion is supported by physiological, pharmacological, and genetic evidence.

First, in addition to root growth inhibition (Fig. 1B–D), it was observed that high NH_4_^+^ up-regulated transcription of ABA biosynthesis genes (Fig. 1A). Subsequent results revealed that high NH_4_^+^ increased ABA content in roots (Fig. 1E). A similar induction was also observed in *A. thaliana* (Li *et al.*, 2012). To unravel the mechanism of ABA action under high-NH_4_^+^ stress, we adopted a pharmacological approach using applications of exogenous ABA and Flu, an inhibitor of ABA biosynthesis (Yoshioka *et al.*, 1998). As expected, the treatment with ABA and Flu resulted in a decrease or increase in the inhibition of root growth induced by high NH_4_^+^ stress, respectively (Fig. 1B–D). Consistent with this, a mutant with higher endogenous levels of ABA, *aba8ox3*, and one with lower levels, *phs3*, were tolerant and sensitive to high-NH_4_^+^ treatment, respectively (Fang *et al.*, 2008; Cai *et al.*, 2015) (Fig. 2A–C). To sum up, our results suggest that ABA plays a significant positive role in the response to high-NH_4_^+^ stress.

Secondly, it has been proposed that a plant’s tolerance to high NH_4_^+^ is related to its capacity for NH_4_^+^ assimilation (Cruz *et al.*, 2006; Liu and von Wirén, 2017). However, our results show that high-NH_4_^+^ stress can inhibit the activities of key enzymes of NH_4_^+^ assimilation, including GS, GOGAT, and GDH in roots; that is, primary NH_4_^+^ assimilation is functionally impaired in roots under high NH_4_^+^ (Fig. 3). Our results also show, however, that high-NH_4_^+^ treatment up-regulates the transcription of genes encoding NH_4_^+^ assimilation enzymes in roots, namely *OsGS1.2* and *OsNADH-GOGAT1*, and that these inductions are enhanced in *aba8ox3* and weakened in *phs3*, while they do not occur in *sapk9* and *bzip20* (Supplementary Figs S4, S5). This shows that differential regulation of NH_4_^+^ assimilation takes place transcriptionally and post-transcriptionally in rice and that the OsSAPK9–OsbZIP20 pathway is involved in this regulation. The results are in agreement with previous reports in *A. thaliana*, showing that the regulation of GS/GOGAT enzymes by NH_4_^+^ can occur differently at levels of transcription, post-transcription, and post-translation (Thomsen *et al.*, 2014; Guan *et al.*, 2016; Jian *et al.*, 2018).

In rice roots, OsGS1.2 and OsNADH–GOGAT1 are expressed abundantly in the surface cell layers of roots, namely in the cells of the epidermis and the exodermis, and this can protect the remainder of the root tissue from high-NH_4_^+^ toxicity (Kronzucker *et al.*, 2001; Ishiyama *et al.*, 2003, 2004; Tabuchi *et al.*, 2007; Hachiya *et al.*, 2012). Gln, the major product of NH_4_^+^ assimilation and the principal organic N form involved in long-distance N transport in the xylem, can be transported to the cortex and the central cylinder (Fukumorita and Chino, 1982; Lea *et al.*, 2007). While ABA biosynthesis in roots mainly occurs in the pericycle (Boursiac *et al.*, 2013), in such cases, once NH_4_^+^ is taken up by the roots, ABA may be rapidly transported to the surface of roots and enhance the catalytic activities of OsGS1.2 and OsNADH–GOGAT1, mediated by the OsSAPK9–OsbZIP20 pathway, protecting roots from the effects of high NH_4_^+^. In line with this assumption, our results show that addition of exogenous ABA in roots can increase GS and GOGAT activities in roots, especially under high-NH_4_^+^ stress, and that the effect of exogenous ABA on GS/GOGAT enzyme activities was weakened or even disappeared (Fig. 9; Supplementary Fig. S2). Furthermore, overexpression of *OsGS1.1* and *OsGS1.2* under the control of the *Cauliflower mosaic virus* (CaMV) 35S promoter resulted in increased GS activities but also increased sensitivity to abiotic stress and poor plant growth, revealing that higher expression of *OSGS1.1* and *OsGS1.2* in all tissues would disturb the normal nutrient metabolism and signaling pathway (Cai *et al.*, 2009). In contrast, co-overexpression of *OsGS1.1/OsGS2* was associated with enhanced tolerance to abiotic stresses and yield improvement (James *et al.*, 2018). These incompatible phenotypes might be due to an imbalance in C/N metabolism (Bao *et al.*, 2014). It is to be expected that the abiotic tolerance and yield improvement resulting from overexpression of *GS* furthermore depends on higher GOGAT activity, sufficient supply of ATP and NADH, and 2-oxoglurate from C metabolism. Taking these results together, manipulating ABA signaling to enhance GS/GOGAT and GDH enzyme activities *in situ* appears a promising strategy to increase NH_4_^+^ assimilation under abiotic stresses.

Thirdly, it is well known that environmental stresses induce oxidative stress and lead to higher accumulation of ROS (Mittler *et al.*, 2011). Moreover, high-NH_4_^+^ treatment has also been reported to augment ROS, especially H_2_O_2_ in roots, pointing to a role for ROS in high-NH_4_^+^-induced root growth inhibition (Liu and von Wiren, 2017; Li *et al.*, 2019). To protect cells from oxidative damage by overaccumulated ROS, plants have evolved a sophisticated antioxidant defense apparatus to scavenge ROS, including enzymatic and non-enzymatic systems (Apel and Hirt, 2004; Mittler *et al.*, 2004). In rice, an OsSE5-mediated antioxidant defense alleviates root elongation from NH_4_^+^ inhibition, indicating that an up-regulated antioxidant apparatus can offset NH_4_^+^ toxicity in roots (Xie *et al.*, 2015). In the past two decades, the crosstalk between ABA and ROS signals has been intensively studied (Gill and Tuteja, 2010). ABA-enhanced stress tolerance has been associated with the induction of antioxidant defense systems, including enzymatic and non-enzymatic components, protecting plant cells against oxidative damage (Zhang *et al.*, 2014; Yang *et al.*, 2015). ABA acts as a key blocker of H_2_O_2_ production during exposure to osmotic stress and increases the activities of antioxidant enzymes against oxidative stress (Lee and Luan, 2012). In our study, the *aba8ox3* mutant, endowed with high endogenous ABA, accumulated less, and the *phs3* mutant, endowed with low endogenous ABA, accumulated more H_2_O_2_ under high-NH_4_^+^ stress (Fig. 4A, B). Consistent with this, the MDA content, as a biomarker of cell membrane injury, was significantly increased in *phs3* compared with its wild type, especially in *osaba8ox3*, indicating that higher ABA can reduce the oxidative damage normally encountered under high-NH_4_^+^ stress (Dey *et al.*, 2016) (Fig. 4C, D). The lower MDA content implies a higher reductive ability, reflecting higher abiotic stress resistance (Marcińska *et al*., 2013). To cope with the overproduction of ROS, plants also employ scavenging enzymes such as SOD, APX, and CAT, to adjust ROS homeostasis (Apel and Hirt, 2004; Mittler *et al.*, 2004; Conde *et al.*, 2011). In our study, high-NH_4_^+^ stress-induced SOD, APX, and CAT activities in *osaba8ox3* were significantly higher than those in the wild type and in *osphs3* (Fig. 4E–J). These results reveal that ABA can enhance the activities of antioxidant enzymes, including SOD, APX, and CAT, followed by reduced ROS accumulation, which results in decreased oxidative damage to roots.

In addition, many plants accumulate compatible solutes for maintaining membrane integrity and scavenging ROS in response to osmotic adjustment (Hong *et al.*, 2000; Couée *et al.*, 2006). Soluble sugars act as osmo-protectants, maintaining cell turgor, and protect the integrity of cell membranes (Couee *et al.*, 2006). Endogenous sugar availability can feed the oxidative pentose phosphate pathway, leading to additional ROS scavenging (Couee *et al.*, 2006). Moreover, exogenous sucrose feeding has been shown to increase SOD, CAT, and APX activities in wheat under salt stress conditions (Yan *et al.*, 2012) and in *A. thaliana* following atrazine treatment (Ramel *et al.*, 2009). Upon NH_4_^+^ stress imposition, higher contents of soluble sugars were accumulated in *aba8ox3* compared with *osphs3* (Supplementary Fig. S6). Another important class of smaller molecules known as ‘compatible osmolytes’ includes proline. Proline has a key role in protecting against oxidative stress by scavenging of ROS (Hong *et al.*, 2000; Sorkheh *et al.*, 2012; Yan *et al.*, 2012). Proline accumulation was closely associated with an increase in ammonium concentration, while high-NH_4_^+^-induced proline accumulation was also involved in regulating antioxidative activity and osmotic adjustment in white clover (*Trifolium repense* L.) (Kim *et al.*, 2004). In our study, higher contents of proline were accumulated in *osaba8ox3* compared with the wild type and *phs3* under high-NH_4_^+^ stress. To sum up, ABA-induced osmolyte accumulation in roots is another possible ABA-mediated resistance mechanism to high NH_4_^+^ (Supplementary Fig. S6). Additionally, GS overexpression also resulted in a reduction of ROS and MDA levels under abiotic stress by enhancing the activities of antioxidant enzymes, including SOD (Lee *et al.*, 2013; Molina-Rueda *et al.*, 2013; Molina-Rueda and Kirby, 2015; James *et al.*, 2018). Glu is required for the synthesis of proline and GSH, while co-overexpression of *OsGS1.1* and *OsGS2* in rice led to greater accumulation of proline and less MDA under drought and salt stresses (James *et al.*, 2018). Mutation of GS in *Locus* plants led to significantly lower proline levels under drought stress (Díaz et al., 2010). Hence, NH_4_^+^ assimilation is clearly linked to antioxidant activity under high-NH_4_^+^ stress, and ABA appears to be involved in this.

### The OsSAPK9–OsbZIP20 pathway is an important positive regulatory system for ABA signaling under NH_4_^+^ stress

ABA-responsive gene expression is directly regulated by TFs that recognize and bind to *cis*-elements in the promoter regions upstream of their target genes (Fujita *et al.*, 2011). bZIP TFs play important roles in the ABA/stress signaling pathway (Kim, 2006; Nijhawan *et al.*, 2008), and these have been designated as ABFs or AREBs (Yamaguchi-Shinozaki and Shinozaki, 2005), but little is known about their functions in rice under high-NH_4_^+^ stress. Of the 13 rice bZIP TF groups, Groups A, C, and S participate in abiotic stress signaling (Nijhawan *et al.*, 2008). Nevertheless, rice has six members in Group C, namely OsbZIP15, OsbZIP20, OsbZIP33, OsbZIP52, OsbZIP58, and OsbZIP88, and, of these, OsbZIP20, OsbZIP33, and OsbZIP88 responded to salt and drought stress, while only OsbZIP20 was induced under high NH_4_^+^, indicating that OsbZIP20 plays a special role under NH_4_^+^ stress (Fig. 5; Supplementary Figs S7, S8) (Nijhawan *et al.*, 2008; Sun *et al.*, 2017). OsbZIP20 is a typical member of the Group C bZIP family, but has no transactivation activity (Fig. 6C), while it can bind the G-box or ABRE (Fig. 6B), which are ubiquitously found in the promoters of plant genes regulated by environmental signals (Nijhawan *et al.*, 2008). Moreover, OsbZIP20 transcript was significantly higher or lower in *aba8ox3* or *phs3* under high-NH_4_^+^ stress, respectively, compared with their wild types (Fig. 5B), while the OsbZIP20 transcript was significantly induced under different high-NH_4_^+^ conditions, suggesting that OsbZIP20 is involved in the response to high NH_4_^+^ via an ABA-dependent pathway (Fig. 5A). Furthermore, the promotion of NH_4_^+^ assimilation and antioxidant enzymes by exogenous ABA was decreased or even disappeared in *bzip20* (Fig. 9). However, when we analyzed the promoters (~2000 bp) of genes encoding NH_4_^+^ assimilation enzymes in roots, there were no bZIP20-binding *cis*-elements. Thus, we conjectured that OsbZIP20 participates in the NH_4_^+^ stress pathway indirectly, by regulating the activities of NH_4_^+^ assimilation and antioxidant enzymes, and the detailed mechanism affecting the enzyme activities of GS/GOGAT by OsbZIP20 should be investigated in future studies. The ABA-dependent phosphorylation of the ABF/AREBs is indispensable for its activation. The ABA-dependent activation of ABF/AREB proteins requires phosphorylation by SnRK2 protein kinases (Furihata *et al.*, 2006; Fujii *et al.*, 2007). In rice, 10 SnRK2 members, designated as OsSAPK1–OsSAPK10 (ABA-activated protein kinase 1–10), have been identified, which are activated by hyperosmotic stress, and Subclass III SnRK2s, including OsSAPK8, 9, and 10, which are also induced by ABA (Kobayashi *et al.*, 2004; Dey *et al.*, 2016). OsSAPK8, OsSAPK9, and OsSAPK10, which possess both autophosphorylation and transphosphorylation activities, control AREB/ABFs in ABA-responsive gene expression under osmotic stress conditions (Fujita *et al.*, 2011; Dey *et al.*, 2016). As OsbZIP20 does not possess transactivation activity and is involved in the ABA response, we analyzed the interaction of OsbZIP20 and OsSAPK8, 9, and 10, which function in response to ABA (Fig. 6C). We demonstrate that only OsSAPK9 can interact with OsbZIP20 *in vitro* and *in vivo*, and that OsSAPK9 also phosphorylated OsbZIP20, and subsequently might activate its function (Fig. 6D–F). Furthermore, when we analyzed the phosphorylation of OsbZIP20 *in vivo*, we found that there were four phosphorylated protein bands in the control condition, and exogenous supply of ABA could induce a new phosphorylated protein band, showing that ABA can further induce the phosphorylation of OsbZIP20 (Fig. 7B). Moreover, this new induced phosphorylated protein band disappeared in the *sapk9* mutant, revealing the role of OsSAPK9 in ABA-induced phosphorylation of OsbZIP20 (Fig. 7B). Previous work had shown that OsSAPK9 is located in the cytosol and the nucleus, similar to other SnRK2 family members (Mao *et al.*, 2010; Tian *et al.*, 2013; Dey *et al.*, 2016), and we also detected the nuclear localization signal (NLS) in the OsbZIP20 amino acid sequence, indicating nuclear localization of OsbZIP20 and suggesting that the interaction between OsSAPK9 and OsbZIP20 occurs in the nucleus. However, a significant signal was also detected in the cytosol, as shown in Fig. 6E, indicating that the interaction between OsSAPK9 and OsbZIP20 may also occur in the cytosol. Another working hypothesis is that OsSAPK9 phosphorylates and activates OsbZIP20 in the cytosol, and then the activated OsbZIP20 is transferred to the nucleus to regulate the transcription of downstream genes involved in acclimation to environmental stress. A similar phenomenon has been reported in previous studies, showing that phosphorylation was responsible for light-modulated GBF2 translocation from the cytosol to the nucleus (Harter *et al.*, 1994; Terzaghi *et al.*, 1997). Clearly, future studies will have to be designed to unravel the possible role of phosphorylation of OsbZIP20 by OsSAPK9 and whether a transfer of activated OsbZIP20 to the nucleus takes place. Our results also suggest that ABA can induce the expression of OsbZIP20 and that OsSAPK9 is involved in this process. Taken together, our data suggest that the initiation of the interaction between OsSAPK9 and bZIP20 can occur without ABA or NH_4_^+^, but that exogenous ABA can significantly enhance the transcription of SAPK9 and bZIP20 and phosphorylation of bZIP20.

In addition, OsSAPK9 was induced by high NH_4_^+^, and the induction was further intensified by applications of endogenous ABA (Supplementary Fig. S9). OsSAPK9 has also been reported to phosphorylate OsbZIP23 in response to drought stress, indicating that plants employ different OsSAPK–OsbZIP groups to deal with high-NH_4_^+^ stress versus osmotic stress. The knockout mutant *sapk9* displayed decreased activities of NH_4_^+^ assimilation and antioxidant enzymes, and higher free NH_4_^+^ and H_2_O_2_ levels in roots, resulting in more oxidative damage to roots (Figs 8, 9; Supplementary Fig. S3). Moreover, ABA-induced NH_4_^+^ assimilation and antioxidant activity enhancement decreased or even disappeared in *sapk9* under high-NH_4_^+^ stress (Figs 8, 9), indicating a strong role for OsSAPK9 in ABA-dependent NH_4_^+^ resistance. Furthermore, the increased or decreased antioxidant activity was also found in overexpression and knockdown lines of *OsSAPK9* under drought stress (Dey *et al.*, 2016), implying that OsSAPK9-mediated antioxidant activity augmentation is a more general mechanism in response to stress. However, enhanced NH_4_^+^ assimilation capacity might be unique to NH_4_^+^ stress. Taken together with previous findings, these observations establish that ABA-dependent phosphorylation of OsbZIP20 by OsSAPK9 protein kinases is involved in the activation of AREB/ABFs in rice under high-NH_4_^+^ stress. Thus, the SnRK2–AREB/ABF system contributes to the presence of a high-NH_4_^+^ adaptive stress response in rice.

The activated ABF/ABRE system can induce the expression of LEA genes, which are novel ABRE-dependent AREB/ABF target genes, and these play crucial roles in cellular tolerance in response to abiotic stress (Battaglia *et al.*, 2008; Yoshida *et al.*, 2010). The expression of *OsLEA* genes can be induced by the application of ABA and by various abiotic stresses (Duan and Cai, 2012). There are 34 *OsLEA* genes in the rice genome, while only 16 of them are induced by exogenous ABA (Wang *et al.*, 2007). As shown in Supplementary Fig. S10, upon imposition of high-NH_4_^+^ stress, much higher expression levels of these genes are induced in *osaba8ox3* compared with the wild type and *phs3*. The up-regulated *OsLEA* genes belonged to group 3, which has been suggested to function in the preservation of protein and membrane structure during various abiotic stresses (Duan and Cai, 2012). Hence, the regulation of the OsLEA3 group by OsSAPK9 and OsbZIP20 warrants further study in the future.

**Fig. 10.**
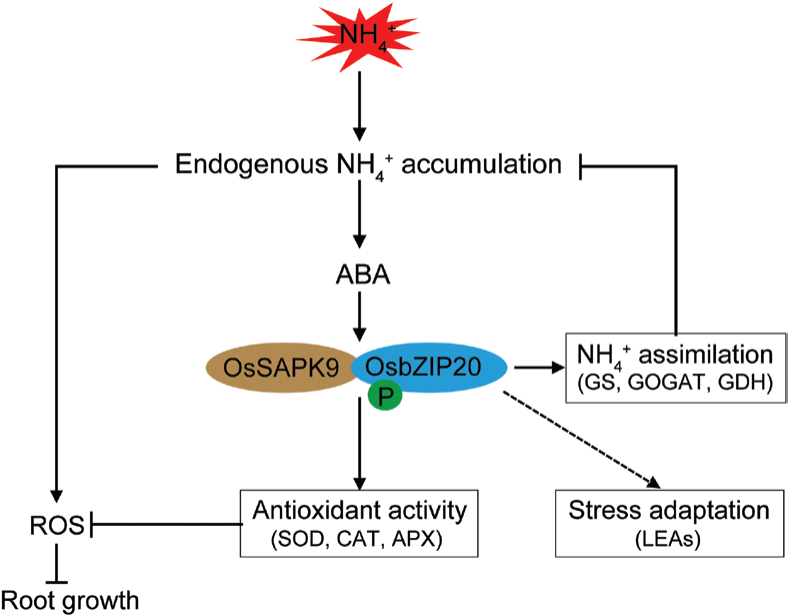
Proposed working model linking ABA signaling in rice roots to the response to high-NH_4_^+^ stress. Under HA stress, OsSAPK9–OsbZIP20 mediated ABA signaling via three routes to relieve HA inhibition of root growth. First, ABA strengthens NH_4_^+^ assimilation by increasing GS, GOGAT, and GDH enzyme activities, reducing free NH_4_^+^ accumulation in roots. Secondly, ABA fortifies the antioxidant apparatus by increasing the enzyme activities of SOD, CAT, and APX, reducing ROS levels and oxidative stress damage. Thirdly, the transcription factor OsbZIP20 up-regulates LEAs to facilitate adaptation to HA stress.

In conclusion, our study shows that the increase of antioxidant enzyme activities and of NH_4_^+^ assimilation are active responses to a high-NH_4_^+^ challenge and are associated with higher endogenous ABA. Moreover, in the process, the *OsSAPK9–OsbZIP20* pathway plays a critical regulatory role (Fig. 10). The understanding of the tolerance mechanism to high NH_4_^+^ involving the *OsSAPK9–OsbZIP20* pathway induced by higher endogenous ABA is of great importance to confronting this significant agronomic problem.

## Supplementary data

Supplementary data are available at *JXB* online.

Fig. S1. Relative transcription of *ABA8OX3* in *aba8ox3* and *CRTISO* in *phs3*.

Fig. S2. Exogenous ABA and an ABA inhibitor alter the activities of NH_4_^+^ assimilation enzymes.

Fig. S3. Glu and Gln contents in *sapk9* and *bzip20* mutants after high-NH_4_^+^ treatment.

Fig. S4. Transcript expression analysis of genes encoding NH_4_^+^ assimilation enzymes by qRT-PCR in *aba8ox3* and *phs3* mutants.

Fig. S5. Transcript expression analysis of genes encoding NH_4_^+^ assimilation enzymes by qRT-PCR in *sapk9* and *bzip20* mutants.

Fig. S6. Proline and soluble sugar contents of roots in *osaba8ox3* and *osphs3* under high NH_4_^+^.

Fig. S7. Sequences analysis of OsbZIP20 with OsbZIP52 and AtbZIP9.

Fig. S8. Phosphorylation site prediction in OsbZIP20 by the GPS 2.1 program.

Fig. S9. *OsSAPK9* is involved in the response to high NH_4_^+^.

Fig. S10. Relative transcript levels of *LEA* genes in *aba8ox3* and *phs3* under normal and high-NH_4_^+^ conditions

Table S1. Primers used in this study

eraa076_suppl_Supplementary_Figure_S1_S10

eraa076_suppl_Supplementary_Table_S1
