# Supplementary material for: Endogenous ABA alleviates rice ammonium toxicity by reducing ROS and free ammonium via regulation of the SAPK9–bZIP20 pathway
Source: J Exp Bot. 2020 Feb 17;71(15):4562–77. doi: 10.1093/jxb/eraa076 (PMC7475098; doi:10.1093/jxb/eraa076)
Supplement: eraa076_suppl_Supplementary_Figure_S1_S10 [file eraa076_suppl_supplementary_figure_s1_s10.pdf]

Endogenous ABA Alleviates Rice Ammonium Toxicity by Reducing ROS and Free Ammonium via Regulation of the SAPK9-bZIP20 Pathway

Li Sun[1,2#], Dong-Wei Di[1#], Guangjie Li[1], Herbert J. Kronzucker[3], Xiangyu Wu[4], and Weiming Shi [1 *]

[1]State Key Laboratory of Soil and Sustainable Agriculture, Institute of Soil Science, Chinese Academy of Sciences, No.71 East Beijing Road, Nanjing, Jiangsu 210008, China

[2]State Key Lab of Crop Genetics and Germplasm Enhancement, Cytogenetics Institute, Nanjing Agricultural University/JCIC-MCP, Nanjing, Jiangsu 210095, China

[3]School of Agriculture and Food, University of Melbourne, Parkville, VIC 3010, Australia & Faculty of Land and Food Systems, University of British Columbia, Vancouver, BC, V6T 1Z4 Canada

[4]Key Lab of Plant-Soil Interaction, MOE, College of Resources and Environmental Sciences, China Agricultural University, 100193 Beijing, China

[#] These authors contribute equally to this work.

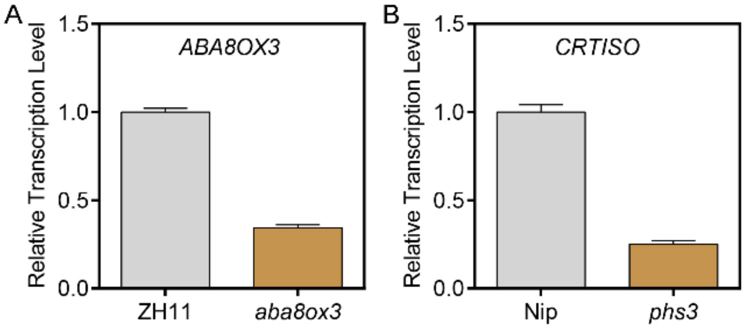

Fig. S1. Relative transcription of *ABA8OX3* in *aba8ox3* and *CRTISO* in *phs3*

Roots of five-day-old seedling were collected for RNA extraction and qPCR analysis. Data are the means of three biological replicates.

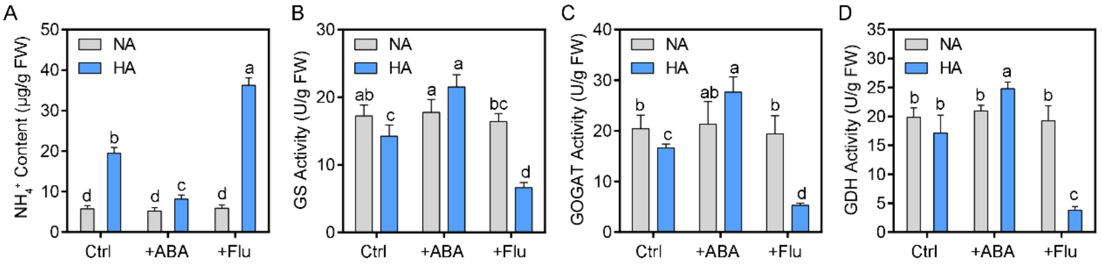

Fig. S2. Exogenous ABA and ABA inhibitor alter the activities of NH_4^+-assimilation enzymes

(A-D) NH_4^+ contents (A); GS activity (B); GOGAT activity (C), and GDH activity (D) of *aba8ox3*, *phs3*, and their backgrounds ZH11 and Nip, grown in normal-NH_4^+ (NA) and high-NH_4^+ (HA) medium. Five-day-old seedlings were transferred to treatment medium for another ten days. Data are analyzed by two-way ANOVA following Duncan's test (n=3). Error bars with different letters represent a statistical difference ($P < 0.05$, Duncan's test).

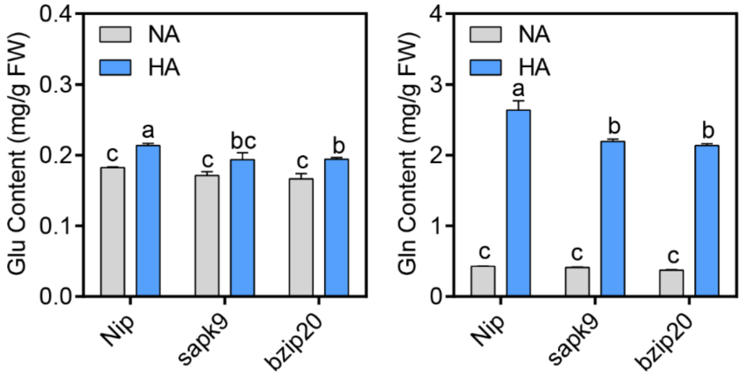

Fig. S3. Glu and Gln contents in *sapk9* and *bzip20* mutant after high-NH_4^+ treatment

Five-day-old seedling were transferred to HA medium for another ten days, and then roots were collected for Glu and Gln determination. Data are the means of three biological replicates. Data are analyzed by two-way ANOVA following Duncan's test. Error bars with different letters represent a statistical difference ($P < 0.05$, Duncan's test).

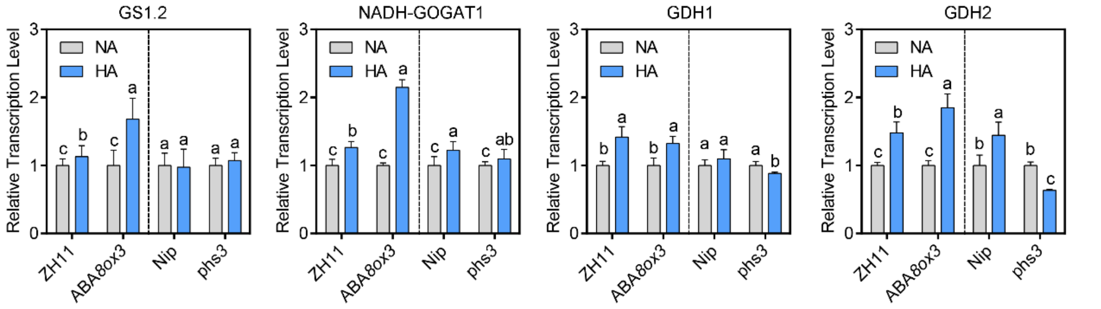

Fig. S4. Transcriptional analysis of genes encoding NH_4^+ assimilation enzymes by qRT-PCR in ABA8*ox3* and *phs3* mutants

The regulation of NH_4^+ assimilation related genes in rice under HA treatment in ABA8*ox3* and *phs3* mutants; five-day-old seedling were transferred to HA medium for another 12 hours, and then roots were collected for RNA extraction and qPCR analysis. Data are the means of three biological replicates. Data are analyzed by two-way ANOVA following Duncan's test. Error bars with different letters represent a statistical difference ($P < 0.05$, Duncan's test).

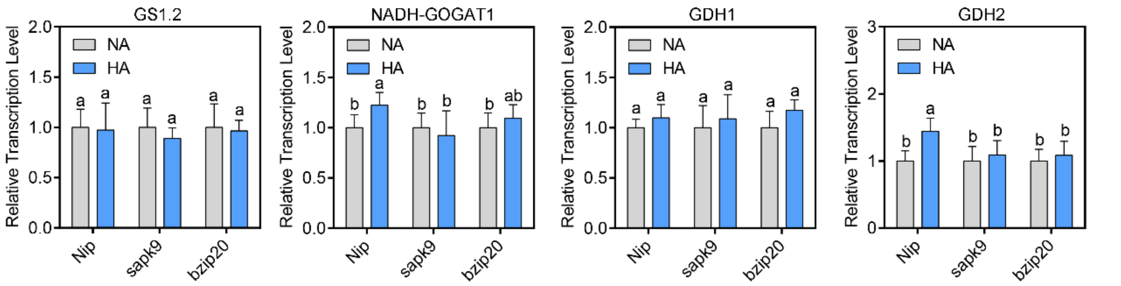

Fig. S5. Transcriptional analysis of genes encoding NH_4^+ assimilation enzymes by qRT-PCR in *sapk9* and *bzip20* mutants

The regulation of NH_4^+ assimilation related genes in rice under HA treatment in *sapk9* and *bzip20* mutants; five-day-old seedling were transferred to HA medium for another 12 hours, and then roots were collected for RNA extraction and qPCR analysis. Data are the means of three biological replicates. Data are analyzed by two-way ANOVA following Duncan's test. Error bars with different letters represent a statistical difference ($P < 0.05$, Duncan's test).

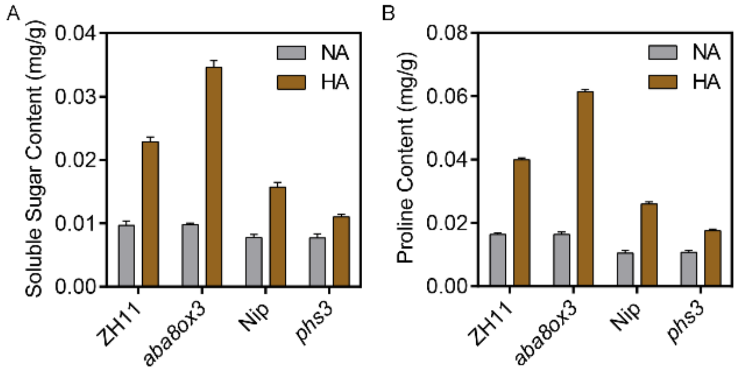

Fig. S6. Proline and soluble sugar contents of root in *osaba8ox3* and *osphs3* under high NH_4^+

Soluble sugar and free proline contents were measured in the roots of plants germinating under control solution for five days and then subjected to high-NH_4^+ conditions for another 10 days. Values are the means ± SD (n = 3). FW, fresh weight.

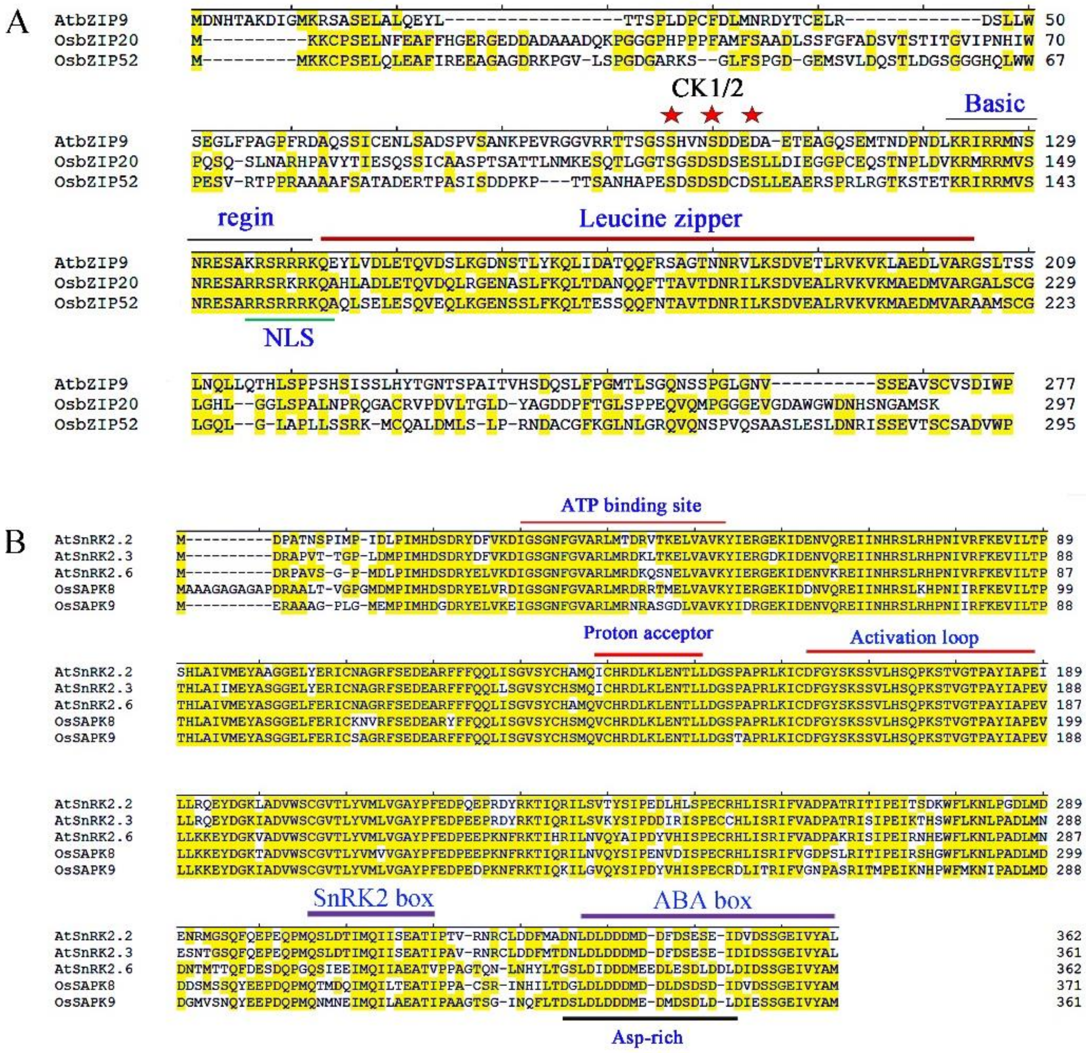

Fig. S7. Sequences analysis of OsbZIP20 with OsbZIP52 and AtbZIP9

Amino acid sequence alignment of OsbZIP20 with OsbZIP52 and AtbZIP9 protein for the prediction of OsbZIP20 secondary structure following the PROSITE ExPASy bioinformatics tool.

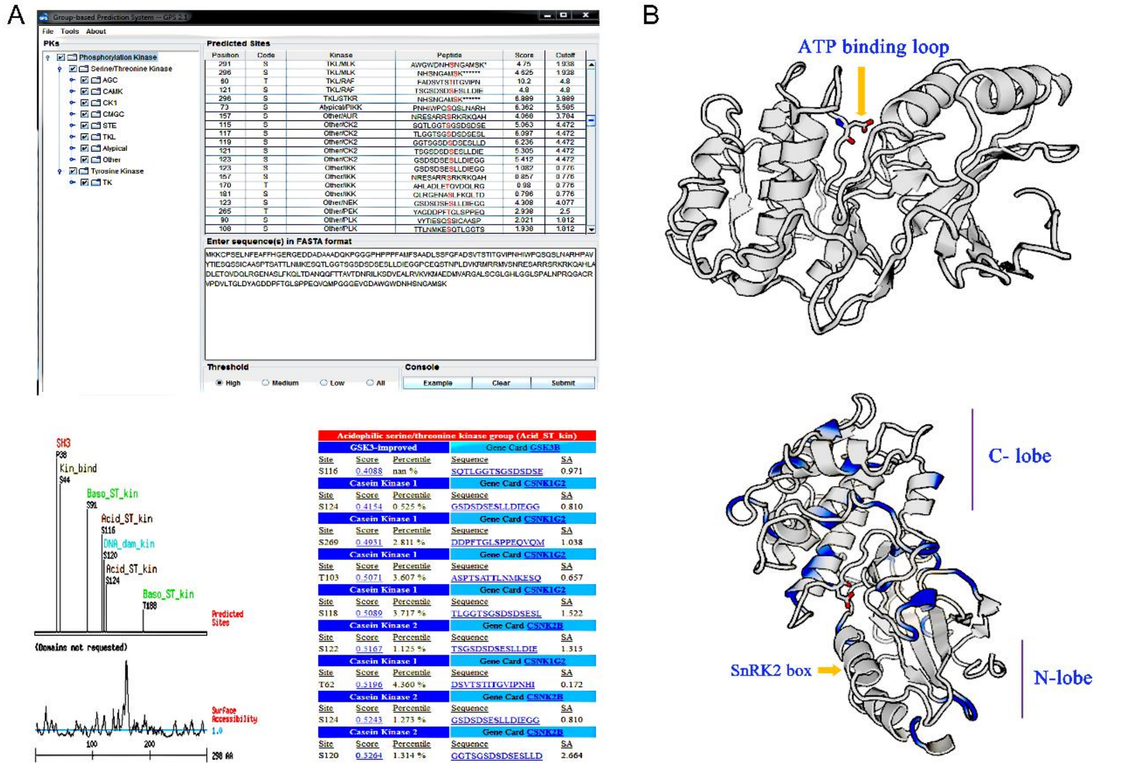

Fig. S8. Phosphorylation site prediction in OsbZIP20 by the GPS 2.1 program

(A) and the Scansite Motif Scanner online server (http://scansite.mit.edu/); (B) The protein structure of OsbZIP20 with SWISS- MODEL online server. (https://swissmodel.expasy.org), using Arabidopsis SnRK2.6 as a template. The SnRK2 box, ATP binding loop and the activation loop segments are highlighted, respectively.

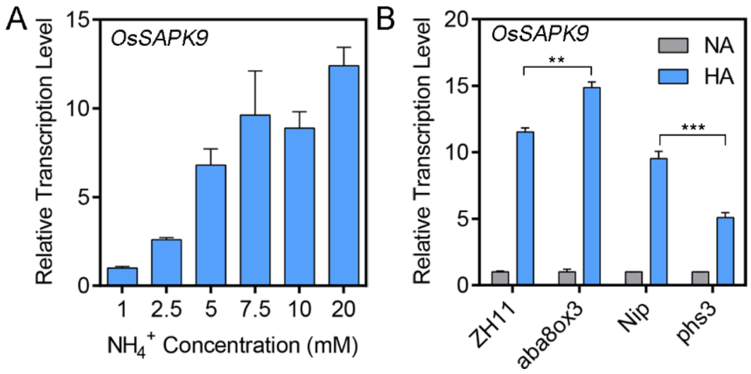

Fig. S9. *OsSAPK9* is involved in the response to high NH_4^+

(A) Transcript levels of *OsSAPK9* in Nip under different concentrations of NH_4^+; five-day-old seedling were transferred to media containing different concentrations of NH_4^+(1, 2.5, 5, 7.5, 10 and 20 mM) for another 12 hours, and then roots were collected for RNA extraction and qPCR analysis. (B) Transcript levels of *OsSAPK9* in *aba8ox3*, *phs3*, and their backgrounds ZH11 and Nip under HA conditions. Five-day-old seedling were transferred to NA and HA media for another 12 hours, and then roots were collected for RNA extraction and qPCR analysis. Data are the means of three biological replicates; Error bars indicate ± SD. $^{**}P < 0.01$ and $^{***}P < 0.001$ (t-test).

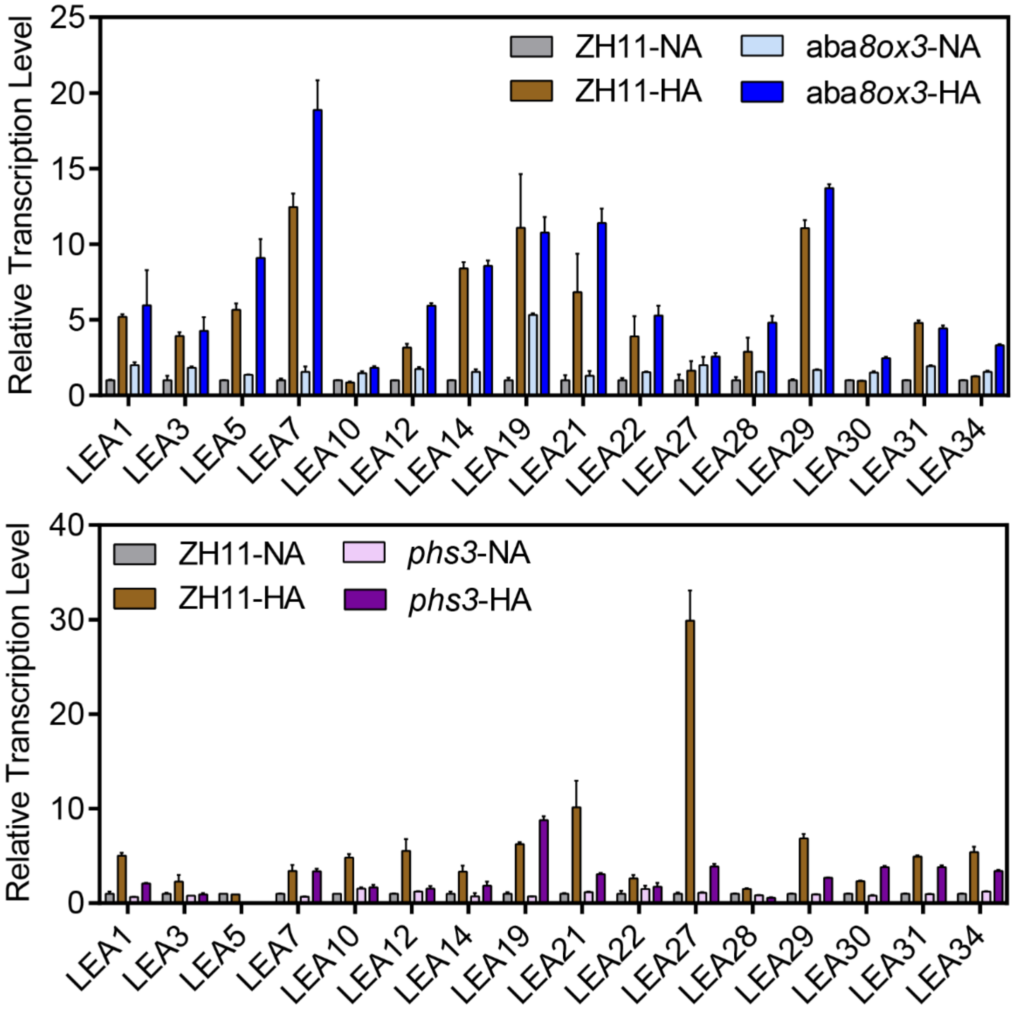

Fig. S10. Relative transcript levels of *OsLEAs* in *aba8ox3* and *phs3* under normal and high NH_4^+ conditions

Five-day-old seedling were transferred to HA medium for another 12 hours, and then roots were collected for RNA extraction and qPCR analysis. Data are the means of three biological replicates.
